# Early Alzheimer’s Disease Screening Approach Using Plasma Biomarkers

**DOI:** 10.3390/ijms241814151

**Published:** 2023-09-15

**Authors:** Lourdes Álvarez-Sánchez, Carmen Peña-Bautista, Laura Ferré-González, Laura Cubas, Angel Balaguer, Bonaventura Casanova-Estruch, Miguel Baquero, Consuelo Cháfer-Pericás

**Affiliations:** 1 Alzheimer Disease Research Group, Health Research Institute La Fe, 46026 Valencia, Spain; lourdes_alvarez@iislafe.es (L.Á.-S.); mariadelcarmen_pena@iislafe.es (C.P.-B.); laufegon@alumni.uv.es (L.F.-G.); baquero_miq@gva.es (M.B.); 2 Division of Neuroinmunology, University and Polytechnic Hospital La Fe, 46026 Valencia, Spain; laura_cubas@iislafe.es (L.C.); casanova_bon@gva.es (B.C.-E.); 3 Math Faculty, Universitat de València, 46026 Valencia, Spain; angel.balaguer@uv.es

**Keywords:** Alzheimer’s disease, frontotemporal dementia, plasma, biomarker, SIMOA, diagnosis

## Abstract

Alzheimer’s disease (AD) is the most prevalent dementia, but it shows similar initial symptoms to other neurocognitive diseases (Lewy body disease (LBD) and frontotemporal dementia (FTD)). Thus, the identification of reliable AD plasma biomarkers is required. The aim of this work is to evaluate the use of a few plasma biomarkers to develop an early and specific AD screening method. Plasma p-Tau181, neurofilament light (NfL), and glial fibrillary acid protein (GFAP) were determined by Single Molecule Assay (SIMOA^®^ Quanterix, Billerica, MA, USA) in patients with mild cognitive impairment due to AD (MCI-AD, *n* = 50), AD dementia (*n* = 10), FTD (*n* = 20), LBD (*n* = 5), and subjective cognitive impairment (SCI (*n* = 21)). Plasma p-Tau181 and GFAP showed the highest levels in AD dementia, and significant correlations with clinical AD characteristics; meanwhile, NfL showed the highest levels in FTD, but no significant correlations with AD. The partial least squares (PLS) diagnosis model developed between the AD and SCI groups showed good accuracy with a receiver operating characteristic (ROC) area under curve (AUC) of 0.935 (CI 95% 0.87–0.98), sensitivity of 86%, and specificity of 88%. In a first screen, NfL plasma levels could identify FTD patients among subjects with cognitive impairment. Then, the developed PLS model including p-Tau181 and GFAP levels could identify AD patients, constituting a simple, early, and specific diagnosis approach.

## 1. Introduction

Alzheimer’s disease (AD) is the main cause of dementia, and it has a high impact on society [1]. It is estimated that there are 50 million people with dementia worldwide, and it is expected to increase to 300 million people by 2050 due to the aging population [2]. Also, AD has a long preclinical/prodromal period. In this sense, it is important to develop markers that could identify those patients with a high risk of developing dementia.

Regarding physiopathological mechanisms of the disease, AD is characterized by extracellular deposition of amyloid-β-forming plaques and intracellular deposition of Tau-forming neurofibrillary tangles. Both molecules are present in the brain tissue, and the cause of the change from a normal soluble form to a non-soluble form is still unknown. The most accepted theory is the amyloid cause, in which the amyloid metabolism is affected, causing the production of amyloid-β42 (Aβ42) oligomers, the aggregation of fibrils, and the formation of plaques. Moreover, this process is responsible for the neurodegenerative cascade. Abnormal hyperphosphorylated tau proteins on different amino acid residues (p-Tau at Thr181, 217, 231, etc.) self-aggregate in neurons and form neurofibrillary tangles, which impair axonal transport and lead to synaptic dysfunction and neuronal death. Also, inflammatory responses to the brain injury and oxidative stress increase [3]. A synergic effect between amyloid-β plaques and Tau tangles was described in the process of neurodegeneration [4]. However, the levels of Aβ42 increased during the preclinical and prodromal stage, while they remain stable during the development of dementia [5]. On the other hand, the levels of p-Tau increased in the prodromal stages, but they were more linked to the Tau pathology in AD (extension of Tau tangles across the brain) [6]. Thus, Tau could be a helpful biomarker of the degenerative process [7]. 

These alterations can be detected from cerebrospinal fluid (CSF) biomarkers or the PET-amyloid technique [8]. However, this diagnostic method is based on invasive and expensive techniques. In this context, plasma biomarkers could constitute a promising AD diagnosis approach. The detection of the core AD biomarkers (Aβ42, t-Tau, and p-Tau181) showed some problems because of the low concentration of these molecules in blood [9]. The high sensitivity of new technology (SIMOA^®^) [10] could improve the diagnosis accuracy and the discrimination between AD and other dementias [11,12]. In fact, plasma biomarkers could be altered since early AD stages, even in the presymptomatic stage [13]. 

Recent research has focused on the measurement of the classic biomarkers of amyloidosis (Aβ42 and Aβ42/Aβ40 ratio in plasma) [14,15,16]. Actually, some works showed that phosphorylated tau (p-Tau) is a promising plasma biomarker of AD [17,18,19]. Moreover, the combination of p-Tau and neurofilament light chain (NfL) provided satisfactory results for early AD diagnosis [20,21]; in addition, a previously developed model including Aβ42, Aβ42/Aβ40 ratio, t-Tau, p-Tau181, and NfL [22] showed promising results in the early diagnosis of AD. 

Regarding the abnormal astroglia activation response as a pathological pathway involved in neurodegenerative diseases, the glial fibrillary acidic protein (GFAP) plasma biomarker has been studied in Alzheimer’s disease [23,24,25,26]. GFAP is an astrocytic cytoskeletal protein, and its plasma levels could increase due to abnormal astrocytic functional remodeling (astrogliosis) [27]. Some research in animal and cell models showed that the reactive astrocytes could penetrate and surround amyloid plaques, possibly contributing to the amyloid deposition process [28,29].

In general, the interest in early and minimally invasive AD diagnosis methods has increased even more after the approval of the Lecanemab-Irmb^®^ drug, which is the first modifier treatment for AD with greater effectiveness in early AD stages [30]. Therefore, there is a high unmet need in the development of easily accessible biomarkers to be applied to the general population to identify patients that could potentially benefit from treatment. The current disadvantages of plasma biomarkers are the high technology costs, the lack of reliable cut-off levels, and the low AD specificity among other dementias.

Among the dementias that are clinically similar to AD, frontotemporal lobar degeneration (FTD) is the most prevalent dementia. It is associated with the degeneration of the frontal and temporal lobes [3]. The recent diagnostic criteria included the behavior variant (bvFTD) and primary progressive aphasia (PPA), which included the semantic variant (svFTD) and non-fluent aphasia (PNFA) [31,32]. In general, FTD is one of the most common causes of early-onset dementia, with a similar frequency to AD in patients younger than 65 years old [33,34]. The neurodegeneration causes are still unknown, but approximately 90% of cases are related to Tau deposition and TAR DNA-binding protein (TDP-43) [35]. The FTD syndrome could occasionally manifest as an AD-like amnestic syndrome, mostly in older patients [36]. Another prevalent dementia is Lewy body dementia (LBD). The neuropathological changes observed in the brain are cortical and brainstem Lewy body deposits. However, spongiform changes, neurofibrillary tangles, and similar changes as those observed in AD are very frequent [3]. Therefore, the identification of specific biomarkers to distinguish AD from other dementias is required [37].

The hypothesis of this work is that the combination of a few neurodegeneration biomarkers in plasma (p-Tau181, NfL, and GFAP) could provide high sensitivity and specificity for the early diagnosis of AD (defined as mild cognitive impairment (MCI-AD)), as well as discriminating between early AD and other neurocognitive diseases (FTD and LBD).

## 2. Results and Discussion

### 2.1. Demographic and Clinical Description of Participants

The participants’ demographic and clinical characteristics are summarized in Table 1. As can be seen, no statistically significant differences were observed among groups for sex; while statistically significant differences were observed for age and educational levels (*p* < 0.05). Specifically, these differences were observed between MCI-AD and subjective cognitive impairment (SCI), between SCI and FTD, and between AD (MCI and dementia) and FTD. For the apolipoprotein E (ApoE) genotype, a statistically significant difference was obtained for the ε4-carrier prevalence among all the clinical groups. Also, neuropsychological tests (MMSE, RBANS, CDR) showed significant differences among groups. 

For CSF biomarkers, Aβ42 showed statistically significant lower levels in the MCI-AD group than in the SCI, FTD, and LBD groups; p-Tau181 showed higher levels in the MCI-AD group, followed by the LBD group, and the FTD group; t-Tau showed higher concentrations in the MCI-AD group, followed by LBD and FTD groups, and the lowest values were obtained in the SCI group; NfL showed higher levels in the FTD group, followed by the MCI-AD group, LBD group, and SCI group.

Regarding genetic data, only some FTD patients (65%) were evaluated for the most common cause of genetic FTD (mutations in *C9ORF72*, granulin (*GRN*) genes). From them, 30% did not show any mutation, 10% showed the *C9ORF72* hexanucleotide expansion, 10% showed mutation in GRN, and 15% showed a mutation of uncertain meaning.

### 2.2. Plasma Biomarkers

The results obtained for the plasma biomarkers determined in each participant group are summarized in Table 2. The plasma p-Tau181 concentration was measured in the AD (MCI and dementia) and the SCI groups, showing significant differences among them. The highest p-Tau181 levels were obtained in the AD dementia group.

The plasma NfL levels were measured in all the participants groups, obtaining the highest levels in the FTD group and the lowest levels in the SCI group. Specifically, significant differences were obtained between SCI and MCI-AD (*p* < 0.01), SCI vs. AD dementia groups (*p* < 0.01), SCI vs. FTD (*p* < 0.01), and MCI-AD vs. FTD (*p* < 0.01).

The plasma GFAP levels were measured in all the participants’ groups. The highest levels were obtained in the AD dementia group and the lowest in the SCI group. Specifically, significant differences were obtained between the SCI group and each other group (MCI-AD (*p* < 0.01), AD dementia (*p* < 0.01), FTD (*p* < 0.05), LBD (*p* < 0.03)). Also, statistically significant differences were obtained between the AD group (MCI and dementia) and FTD (*p* < 0.01); and between AD (MCI and dementia) and non-AD participants (FTD, LBD, SCI) (*p* < 0.01). In addition, significant differences were found between MCI-AD and FTD groups.

**Figure 1:**
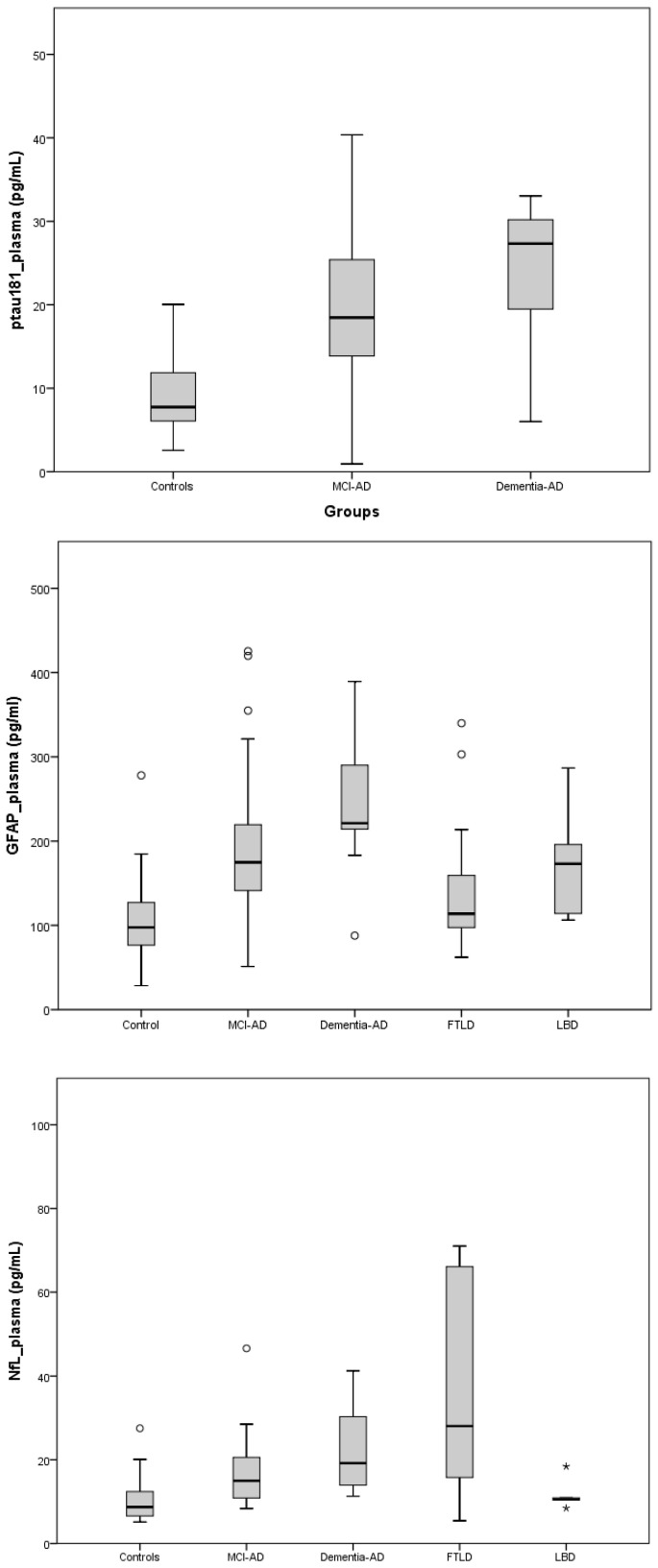
Boxplots representing the plasma biomarker levels obtained for each participant group. The circles represent slight outliers (q < Q1 − 1.5 IQR or q > Q3 + 1.5 IQR), and * represents extreme outliers (q < Q1 − 3 IQR or q > Q3 + 3 IQR).

Figure 1 shows the boxplots for each plasma biomarker level in the different participants’ groups. As can be seen, GFAP showed the highest levels in AD (AD dementia > MCI-AD), followed by the LBD, FTD, and SCI group, while NfL showed the highest levels in the dementia stage (FTD > AD dementia), followed by the MCI-AD, LBD and SCI group.

### 2.3. Clinical Variables Association

The plasma biomarker levels (p-Tau181, GFAP, NfL) for all the participants were evaluated in relation to the clinical variables (age, neuropsychological scores, and CSF biomarkers) (see Table 3).

For age, a positive significant correlation was observed with p-Tau181 (*p* < 0.01) and GFAP (*p* < 0.01) levels. 

Regarding cognitive status, some plasma biomarkers (p-Tau181, NfL, GFAP) showed a significant negative correlation with MMSE (*p* < 0.01, *p* < 0.05, *p* < 0.05, respectively), while they showed a significant positive correlation with CDR total score, (*p <* 0.01, *p <* 0.04, *p <* 0.02, respectively). Also, p-Tau181 showed negative correlations with RBANS-IM (*p <* 0.01), RBANS-DM (*p <* 0.01), RBANS-V/C (*p <* 0.01), RBANS-A (*p <* 0.01), and total score of RBANS (*p <* 0.01). For NfL, negative correlations were obtained with RBANS-DM (*p <* 0.03), RBANS-V/C (*p =* 0.04), and RBANS total score (*p <* 0.02). Finally, GFAP showed a negative correlation with RBANS-IM (*p =* 0.01), RBANS-L (*p =* 0.04), RBANS-DM (*p <* 0.01), and RBANS total score (*p <* 0.01).

For CSF biomarkers (Aβ42, Aβ40, t-Tau, p-Tau181, NfL, Aβ42/Aβ40, t-Tau/Aβ42), plasma p-Tau181 showed statistically significant positive correlations with CSF t-Tau (*p <* 0.02), p-Tau181 (*p <* 0.01), and t-Tau/Aβ42 (*p <* 0.01) levels, while it showed statistically significant negative correlation with the CSF Aβ42 (*p <* 0.01), and Aβ42/Aβ40 (*p <* 0.01) levels. Similarly, plasma GFAP showed positive correlations with CSF t-Tau (*p <* 0.01), p-Tau181 (*p <* 0.01) and t-Tau/Aβ42 (*p <* 0.01), while it showed negative correlations with CSF Aβ42 (*p <* 0.01), and Aβ42/Aβ40 (*p <* 0.01) levels. Also, plasma NfL only showed a statistically significant positive correlation with NfL CSF levels (*p <* 0.01).

For neurodegeneration biomarkers and individual group analysis, no statistically significant correlations were observed between plasma NfL and CSF t-Tau in any case. However, LBD showed a significant negative correlation between plasma NfL and CSF Aβ42 (0.958, *p <* 0.05). Also, the FTD group showed a significant correlation between plasma GFAP and CSF NfL levels (*r* = 0.568, *p <* 0.02), as well as between plasma NfL and CSF NfL (*r* = 0.523, *p* < 0.03).

Among plasma biomarkers, significant positive correlations were found between p-Tau181 and NfL (*p* < 0.01), and GFAP (<0.01) levels, while GFAP did not show a significant correlation with NfL levels.

### 2.4. Development of Diagnosis Model

From the multivariant analysis, PLS models were performed using the plasma biomarkers (p-Tau181, NfL, GFAP), age, and sex as predictor variables and the participants’ group as the response variable, to discriminate between AD and SCI. Specifically, model 1 was developed to identify MCI-AD patients, model 2 for AD dementia patients, and model 3 for all AD cases.

In general, the three models showed VIP scores > 1 for p-Tau181 and GFAP, approximately 1 for NfL and age, and low scores for sex. The coefficients for these variables in each model equation are summarized in Table 4. As observed, the coefficients for age, p-Tau181, GFAP, and NfL were positive for all the developed models. The corresponding cut-off values obtained for each developed model were 0.13 (model 1), 0.0514 (model 2) and 0.2289 (model 3). 

As can be seen in Table 5, the developed model discriminating between MCI-AD and SCI groups (model 1) showed an AUC of 0.925 (CI 95%, 0.85–0.98), a sensitivity of 0.89%, specificity of 0.82%, and accuracy of 0.88%.

**Figure 2:**
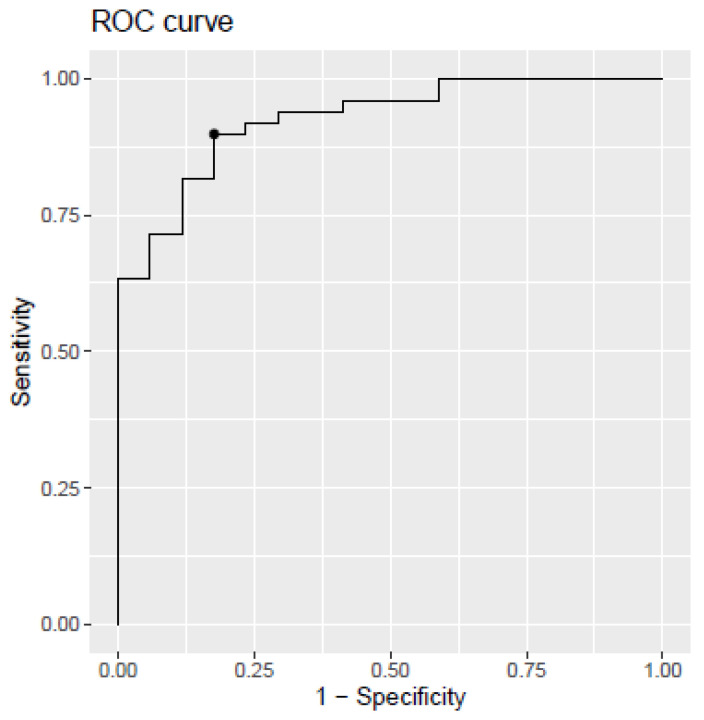
Receiver operating characteristic (ROC) curve for the PLS model developed from plasma biomarkers levels. SCI vs. MCI-AD. The dot within the figure represents the model cut-off, corresponding to the indicated sensitivity and specificity.

The corresponding receiver operating characteristic (ROC) curve analysis was performed and shown in Figure 2.

**Figure 3:**
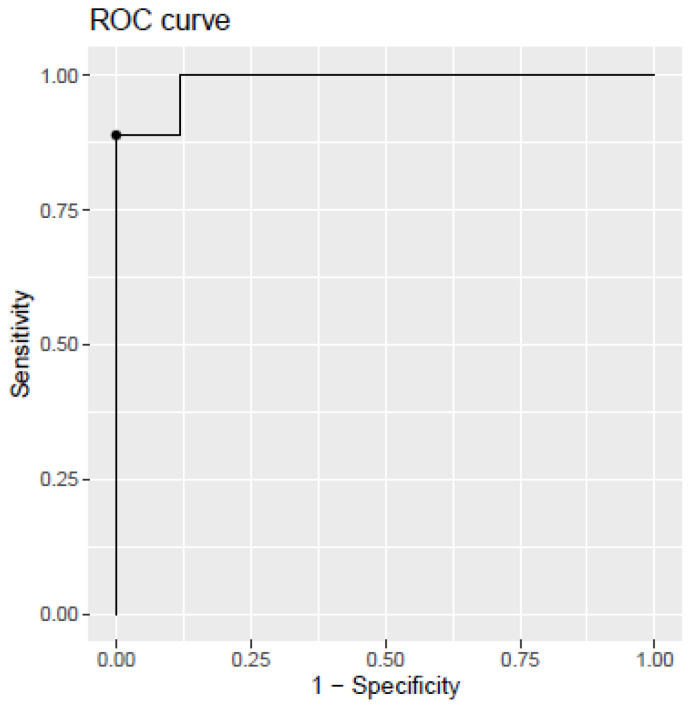
Receiver operating characteristic (ROC) curve for the PLS model developed from plasma biomarkers levels. SCI vs. AD dementia. The dot within the figure represents the model cut-off, corresponding to the indicated sensitivity and specificity.

The developed model discriminating between AD dementia and SCI (model 2) showed an AUC of 0.987 (CI 95%, 0.94–1), a sensitivity of 0.89%, a specificity of 1%, and an accuracy of 0.96%. The corresponding ROC curve is shown in Figure 3. 

**Figure 4:**
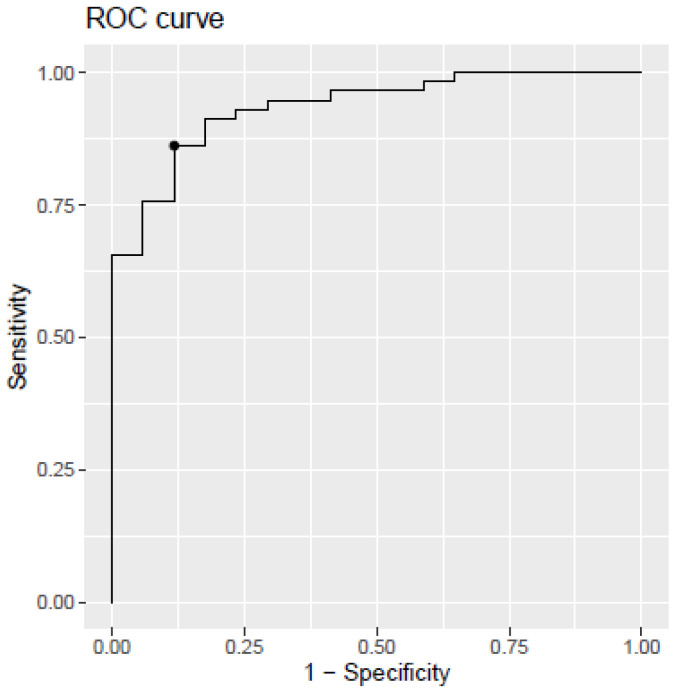
Receiver operating characteristic (ROC) curve for the PLS model developed from plasma biomarkers levels. SCI vs. AD (MCI + dementia). The dot within the figure represents the model cut-off, corresponding to the indicated sensitivity and specificity.

The developed model discriminating between the AD group (MCI and dementia) and SCI (model 3) showed an AUC of 0.935 (CI95%, 0.87–0.98), a sensitivity of 86%, a specificity of 88%, and an accuracy of 87%. The corresponding ROC curve is shown in Figure 4. 

Regarding the specific AD diagnosis, the PLS model developed to discriminate AD (MCI, dementia) from FTD patients showed an AUC of 0.70, high sensibility (80%), but low specificity (20%). The corresponding VIP scores were >1 only for NfL and age.

### 2.5. Discussion

Recent research has focused on the identification of minimally invasive AD biomarkers, especially after the approval of disease-modifying therapies [30,38]. Improving AD diagnosis in the early stages has become more important because of future treatments, whose effectiveness seems to be better in those stages. In this study, some plasma biomarkers have been evaluated to detect early AD. Specifically, they consisted of a small selection of promising biomarkers for AD diagnosis (p-Tau) and neurodegeneration (neurofilament light), in combination with a biomarker of glial activation (GFAP) [39]. 

For plasma p-Tau181, which is the most evaluated isoform with a commercially available kit, higher levels were found in patients with mild dementia (more advanced stage) in comparison with patients with MCI due to AD. Similar results were obtained in previous studies [17,40]. It could be explained by the increase in neurodegeneration, and the increase in cognitive symptoms [41]. Specifically, in AD physiopathology, the p-Tau tangles spread around the brain in the Braak stages showing the progression of the disease [42]. Also, they correlated better with the dementia duration and severity [43]. So, the hypothesis is that the prion-like Tau spread is one of the causes of the cascade of brain destruction. In this sense, the determination of plasma p-Tau could provide relevant biological and clinical information. In the present work, the increased levels of plasma p-Tau181 showed an association with neurocognitive worsening and maybe also with biological worsening. Nevertheless, it is still a challenge that plasma p-Tau levels could reflect the brain Tau spread pathology. 

For plasma NfL, the highest levels were found in FTD patients as in previous studies [44,45]. Also, the levels of NfL were higher in the AD group than in controls. So, plasma NfL could be considered a good biomarker of neurodegeneration [46,47]. In fact, NfL increase could be caused by some neurological pathologies [48,49,50,51] not being AD-specific. In relation to CSF t-Tau levels, considered important neurodegeneration biomarkers, no statistically significant correlations were observed in any participant group. However, the FTD group showed a significant correlation between plasma NfL and CSF NfL levels. In fact, a previous study found that increased NfL plasma levels in FTD were associated with the disease severity and brain atrophy [52]. Therefore, using only NfL to diagnose AD pathology may not be useful, but it could allow a first screening to identify FTD patients among subjects with cognitive impairment.

For plasma GFAP, previous studies showed that the abnormal activation of the glia could play an important role in the development of amyloid burden, constituting a risk factor of developing AD [24,39,53,54]. The present work found that patients diagnosed with AD had the highest concentration of plasma GFAP, even in comparison with other dementias (FTD, LBD), which also show some higher levels than controls. Other studies comparing GFAP levels showed the highest levels in MCI-AD than FTD group. Moreover, they found that the patients who converted to dementia during the follow-up period showed a greater increase in GFAP levels than those without dementia [55]. Also, GFAP could be a biomarker to detect concomitant AD pathology in LBD patients [56]. Specifically, it was observed that patients with α-synuclein pathology and AD neuropathologic changes (confirmed by autopsy) had higher levels of plasma GFAP than patients with α-synuclein pathology and without AD changes. So, GFAP could be associated with brain Aβ and tau burden. Although the only determination of plasma GFAP could not be specific to discriminate AD from other pathologies, it could be useful in other neurological conditions (degenerative and non-degenerative) [57,58,59,60]. 

In the present work, only three plasma biomarkers (p-Tau181, NfL, GFAP) were determined as an early and specific AD diagnosis approach. Satisfactory diagnosis indexes were obtained in the discrimination between early AD and SCI groups. In this way, the newly developed model reduced considerably the analysis cost in comparison with the previous work [22]. It constitutes a relevant advantage for further clinical application to the general population as an early screening method. Previous studies included these plasma biomarkers (p-Tau181, NfL, and GFAP) [61], assessing the association between their levels and the positivity of Aβ by PET [25,62]. Also, these plasma biomarkers were evaluated in a cohort of participants followed over 17 years, and an association between the risk of AD and higher levels of p-Tau181, NfL and GFAP, was observed individually [63]. Moreover, a recent study showed an association between GFAP and Aβ brain deposit (demonstrated by PET) [64]. In the current study, that association was corroborated from CSF biomarkers, which showed significant correlations with GFAP and p-Tau181 plasma biomarkers levels, indicating their relationship with the AD pathology. Also, significant correlations were obtained for neurocognitive scores. In fact, the early AD diagnosis model developed in the present work showed high AUC, sensitivity and specificity, and considered the AD continuum (dementia and MCI). 

Related to specific AD diagnosis, the present study evaluated the plasma biomarkers levels in AD and other neurocognitive disorders. Of note, FTD patients showed the highest NfL plasma levels followed by the AD dementia group. So, NfL could be a useful screening biomarker in early AD stages, differentiating among the two main dementias (AD, and FTD), which share some clinical similarities [65]. Actually, this was corroborated by the absence of significant correlation between NfL and CSF levels of standard AD biomarkers (Aβ42, p-Tau181, t-Tau…). Therefore, NfL could be considered a neurodegeneration biomarker, especially for FTD, but not associated with the specific AD clinical characteristics.

Some correlations between plasma and CSF biomarkers were found. However, determining brain-produced protein in blood is still a challenge. Despite the continuous exchange of molecules between brain and CSF space, only a small proportion of these proteins can cross the blood–brain barrier. Moreover, the measure of these low-concentration plasma proteins could be interfered by high-concentration plasma molecules (e.g., albumin). In this sense, new advances in ultrasensitive analytical techniques (digital immunoassays, and mass spectrometry) constitute an interesting research field. In addition, the possibility that brain-released proteins could be metabolized in the peripheral system (e.g., protease degradation…), and the inter-subject metabolism variability makes it difficult to observe these correlations [66]. 

As a limitation of this study, the sample size is small, especially for the LBD group with only five patients. However, all the participants have a biological verification of the disease through the CSF biomarkers, identifying the AD patients with high accuracy, which is the strength of this work. Moreover, all patients (AD and non-AD) have a protocolized neuropsychological evaluation.

## 3. Materials and Methods

### 3.1. Participants and Samples Collection

A retrospective study based on cross-sectional design has been carried out. The participants were subjects between 50 and 80 years old of both sexes, evaluated in the Cognitive Disorder Unit in the Hospital Universitari I Politècnic la Fe (Valencia, Spain). They completed the diagnosis tests (CSF biomarkers, and neuropsychological evaluation). Specifically, the participants were patients with mild cognitive impairment due to Alzheimer’s disease (MCI-AD, *n* = 50), patients with mild dementia due to Alzheimer’s disease (AD dementia, *n* = 10), patients with frontotemporal lobar degeneration (FTD, *n* = 20), patients diagnosed with Lewy body disease (LBD, *n* = 5), and patients with subjective cognitive impairment (SCI, *n* = 21). The AD diagnosis was established according to the standard criteria of the National Institute of Aging-Alzheimer’s Association (neuropsychological evaluation, and CSF biomarkers) [8]. As exclusion criteria, patients who refused to participate or had significant psychiatric disorders or other comorbidities that could affect cognitive abilities were excluded. Also, patients with severe cognitive impairment due to neurodegenerative diseases were excluded.

The neuropsychological evaluation consisted of the clinical dementia rating scale (CDR), composed of a scale compromising global score (CDR-GS) and the sum of boxes score (CDR-SB) [67,68]; the MMSE (Mini-Mental State Examination) is a scale that evaluates verbal, memory, and visual–constructional skills [69,70]; and the RBANS (Repeatable Battery for the Assessment of Neuropsychological Status) is a 12 test set that evaluates five cognitive domains (RBANS.IM, Immediate Memory; RBANS.V/C, Visuospatial/Constructional; RBANS.L, Language; RBANS.A, Attention; and RBANS.DM, Delayed Memory), which estimate the cognitive decline in patients with AD and other neurodegenerative diseases [71,72,73]. The neuropsychological tests were carried out by accredited neuropsychologists.

CSF samples were obtained from lumbar puncture, following a standard clinical routine. The CSF samples were analyzed by chemiluminescence (CLIA) immunoassay (Lumipulse^®^Fujirebio, Japan) in the clinical diagnosis service from Hospital La Fe. The cut-off values used for each biomarker were (reference level) >830 pg mL^−1^ for Aβ42, <380 pg mL^−1^ for t-Tau, <60 pg mL^−1^ for p-Tau181, >0.069 Aβ42/Aβ40, <0.41 t-Tau/Aβ42, and <810 pg mL^−1^ for NfL [74].

In this sense, the MCI-AD group included participants with alteration in CSF biomarkers (according to the NIA-AA classification) [8], CDR score ≥ 0.5, MMSE score between 24 and 27, and RBANS.DM < 85 (at least 2 altered tests). The AD dementia group included participants with alteration in CSF biomarkers [8], CDR score ≥ 1, and MMSE between 12 and 24, RBANS.DM < 85 (at least 2 altered tests).; these participants were diagnosed with AD in the MCI stage by CSF biomarkers, and a new lumbar puncture was not performed in the current stages.

The FTD group included patients diagnosed according to the International Behavioural Variant FTD Criteria Consortium (FTDC) [31], and classified by PPA and its variants [32]. Actually, 75% of them were diagnosed with bvFTD (*n* = 15), and 25% with PPA (*n* = 5). The LBD group included participants diagnosed according to the requirements of the LBD Consortium [75]. The SCI group included participants without alteration in CSF biomarkers [8], CDR score ≤ 0.5, MMSE ≥ 27, and RBANS.DM ≥ 85; when only one of these tests is impaired, patients are still considered SCI.

Biological samples (blood, and CSF) were collected during routine clinical practice. A venous puncture was performed to obtain the blood samples using a tube containing EDTA; subsequently, the samples were centrifuged for 15 min at 1160× *g* and at room temperature, and the plasma fraction was separated into a new tube. The plasma samples were stored at −80 °C until analysis.

This study was approved by the Ethics Committee from Health Research Institute La Fe (Valencia, Spain) (reference number: 2020-079-1; date: 21 February 2020). All participants signed informed consent prior to their recruitment.

### 3.2. Equipment and Reagents

Quanterix SR-XTM equipment (Billerica, MA, USA), a platform based on SIMOA^®^ technology, was used for the determination of plasma biomarkers levels. In this case, paramagnetic particles are coupled with antibodies designed to bind to specific targets. Specifically, the kits used in this study were SIMOA^®^ human phospho-Tau protein_v2.1, human neurofilament light polypeptide, and human Glial fibrillary acidic protein. They were purchased from Quanterix (Billerica, MA, USA).

### 3.3. Plasma Sample Treatment and Biomarkers Determination

The plasma samples were thawed in ice, and then they were centrifuged for 10 min at 1200× *g* and 4 °C. Plasma biomarkers levels (p-Tau181, GFAP, NfL) were measured using the SIMOA^®^ technology. Briefly, this procedure consisted of sample incubation with magnetic beans, which were conjugated with specific antibodies. Then, a secondary antibody and an enzyme were added, obtaining the immunocomplex (bean/bound protein/ detection antibody). In the lector, each immunocomplex was captured in one individual well, and the lector detected the signal of one single molecule [76]. The plasma biomarkers concentrations were determined from the corresponding calibration curves, constructed from several calibrator points for each assayed peptide.

### 3.4. Statistical Analysis

The SPSS software (IBM SPSS, Inc., Chicago, IL, USA, version 22) was used for the univariate analysis. Numerical variables were expressed as the median and interquartile range (IQR), and the Mann–Whitney and the Kruskal–Wallis tests were used to analyze differences between groups. Categorical variables were expressed as a percentage, and differences between groups were analyzed by the Chi-Square test. For all the analyses, statistical significance was established as *p*-value < 0.05.

The multivariate analysis was performed using R (version 4.2.2), R packages mdatools (version 0.13.1), and cutpoint (version 1.1.2), with IDE R-Studio (version 2022.12.0 Build 353). Partial least squares discriminant analysis (PLS-DA) was built in each case to evaluate the potential of the selected variables and to carry out inferences about the most important ones (VIP scores). The predicted values obtained by Leave-One-Out Cross-Validation were employed in the diagnostic test evaluation. From these corresponding ROC curves and considering the criteria of maximizing the sum sensitivity + specificity, the cut-off for each model was calculated. In addition, AUC, sensitivity and specificity, and their confidence intervals (95%) were estimated by bootstrapping (boot runs = 5000).

## 4. Conclusions

The identification of potential plasma biomarkers (p-Tau181, NfL, GFAP) to improve early and specific AD detection would allow early access to new disease-modifying drugs. Specifically, p-Tau181 and GFAP showed a significant relationship with AD development, while NfL showed general neurodegeneration mainly due to FTD. In this sense, in a first screen, NfL plasma levels could identify FTD patients among subjects with cognitive impairment.

Then, the developed multivariant model (p-Tau181, NfL, GFAP) showed good sensitivity, specificity, and accuracy for early AD diagnosis, constituting a simple diagnosis approach transferable to the general population. Nevertheless, further research will be carried out to validate these preliminary results and establish the corresponding cut-off values.

## Figures and Tables

**Table 1:** Demographic and clinical characteristics for each participants group.

		SCI (*n* = 21)	MCI-AD (*n* = 50)	Mild Dementia-AD (*n* = 10)	FTD (*n* = 20)	LBD (*n* = 5)	*p*-Value (Kruskal–Wallis)
Age (years, median (IQR))		62 (59–70)	71 (68.75–74)	75 (71.25–77)	66.50 (59.75–70.75)	70 (70–73)	<0.01
Sex (% women)		57.1%	56%	80%	65%	40%	>0.05
Educational level (n%)	Primary	28.6%	54%	70%	55%	-	<0.05
	Secondary	23.8%	26%	10%	40%	40%	
	University	47.6%	20%	20%	5%	60%	
ApoE genotype (% ε4 carrier)		9%	38%	30%	0%	20%	<0.05
CSF Aβ42 (pg mL^−1^) (median (IQR))		1043.64 (938.5–1431.5)	509 (450.1–760)	483 (372–674)	1192 (867.75–1669)	819.24 (657–1204.62)	<0.01
CSF p-Tau181 (pg mL^−1^) (median (IQR))		32 (24.5–42.5)	80.5 (58.75–113.5)	98 (71.5–159)	39.5 (24.25–57)	57.5 (34.25–92.75)	<0.01
CSF t-Tau (pg mL^−1^) (median (IQR))		233 (152.5–302.5)	477.5 (328.25–769.5)	768 (432.5–997)	316 (227–549.5)	379.5 (203.75–516.25)	<0.01
CSF Aβ40 (pg mL^−1^) (median (IQR))		10,279 (8882–13,519)	13,217.5 (8545.5–15,064.25)	13,807 (8457–15,054)	12,133 (8770.5–16,159.25)	12,307 (11,964–/)	>0.05
CSF NfL (pg mL^−1^) (median (IQR))		533.71 (443.89–775.17)	1160.52 (825.26–1308.24)	1261.21 (788.26–1717.72)	3019.16 (965.55–4880.87)	948.67 (813.14–/)	<0.01
CSF Aβ42/Aβ40 (median (IQR))		0.107 (0.099–0.114)	0.052 (0.043–0.059)	0.047 (0.043–0.056)	0.107 (0.104–0.115)	0.066 (0.05–/)	<0.01
CSF t-Tau/Aβ 42 (median (IQR))		0.19 (0.16–0.24)	0.72 (0.5–1.7)	98 (71.5–159)	0.27 (0.21–037)	0.46 (0.11–0.46)	<0.01
CDR (median (IQR))		0 (0–0)	0.5 (0.5–0.5)	1 (1–2)	0.5 (0.5–1)	0.5 (0.5–0.75)	<0.01
MMSE (median (IQR))		29 (27.75–30)	26 (23.75–28.25)	17.50 (12.5–27)	23 (20–27)	24 (20.50–27.50)	<0.01
RBANS-IM (median (IQR))		92 (82–100)	69 (60–78.75)	61 (44–69)	59 (47.75–73.75)	65 (52.50–84)	<0.01
RBANS-V/C (median (IQR))		101 (91.25–114.25)	84 (71.25–96)	66 (56–72)	78 (61.5–89)	69 (58.5–82)	<0.01
RBANS-L (median (IQR))		89 (84.25–94.5)	85 (64–96)	57 (51–85)	55.5 (44–69)	87 (69.5–92)	<0.01
RBANS-A (median (IQR))		91 (89.50–100)	73.5 (56–88)	49 (49–56)	67.5 (53 –94.75)	75 (60.5–84.5)	<0.01
RBANS-DM (median (IQR))		100.5 (96.25–109)	69.5 (52–88.5)	44 (40–44)	52 (47–70.5)	78 (57.5–83)	<0.01
Total RBANS (median (IQR))		90.5 (85.75–105.25)	71.5 (56.75–80.25)	50 (45–53)	54 (48–69)	55 (49–74.50)	<0.01

IQR: Inter-quartile range.

**Table 2:** Plasma biomarker levels in each participant group.

Plasma Biomarker (pg mL^−1^, Median (IQR))	SCI (*n* = 21)	MCI-AD (*n* = 50)	Mild Dementia-AD (*n* = 10)	FTD (*n* = 20)	LBD (*n* = 5)	*p*-Value (Kruskal–Wallis)	*p*-Value (Mann–Whitney)
p-Tau181	7.745 (5.97–11.99)	18.47 (13.48–25.43)	27.33 (18.69–30.46)	–	–	<0.01	^0^ <0.01 ^1^ <0.01
NfL	8.72 (6.51–12.59)	14.98 (10.82–20.84)	19.18 (12.75–33.34)	28.04 (15.37–67.23)	10.52 (9.42–14.66)	<0.01	^0^ <0.01 ^1^ <0.01 ^2^ <0.01 ^3^ >0.05 ^4^ >0.05 ^5^ >0.05 ^6^ <0.01
GFAP	97.53 (75.6–132.01)	174.92 (141.1–221.93)	221.32 (198.73–291.42)	113.76 (96.93–162.89)	173.16 (110.26–241.37)	<0.01	^0^ <0.01 ^1^ <0.01 ^2^ <0.05 ^3^ <0.03 ^4^ <0.01 ^5^ <0.01 ^6^ <0.02

^0^ = SCI vs. MCI-AD; ^1^ = SCI vs. AD dementia; ^2^ = SCI vs. FT; ^3^ = SCI vs. LBD; ^4^ = AD (MCI and dementia) vs. FTD; ^5^ = AD (MCI and dementia) vs. non-AD (SCI, FTD, LBD); ^6^ = MCI-AD vs. FTD.

**Table 3:** Correlations between plasma biomarkers levels and clinical characteristics for all the participants.

	Plasma p-Tau181 (pg mL^−1^) Pearson (*r* (*p*-Value))	Plasma NfL (pg mL^−1^) Pearson (*r* (*p*-Value))	Plasma GFAP (pg mL^−1^) Pearson (*r* (*p*-Value))
Age	0.322 (<0.01) *	0.008 (>0.05)	0.389 (<0.01) *
MMSE	−0.459 (<0.01) *	−0.198 (<0.05) *	−0.2 (<0.05) *
CDR	0.367 (<0.01) *	−0.214 (<0.04) *	0.238 (<0.02) *
RBANS-IM	−0.518 (<0.01) *	−1.76 (>0.05)	−0.264 (=0.01) *
RBANS-L	−0.179 (>0.05)	−0.183 (>0.05)	−0.211 (=0.04) *
RBANS-DM	−0.51 (<0.01) *	−0.231 (<0.03) *	−0.323 (<0.01) *
RBANS-V/C	−0.405 (<0.01) *	−0.214 (=0.04) *	−0.185 (>0.05)
RBANS-A	−0.322 (<0.01) *	−0.179 (>0.05)	−0.133 (>0.05)
TOTAL RBANS	−0.520 (<0.01) *	−0.248 (<0.02) *	−0.324 (<0.01) *
CSF Aβ40 (pg mL^−1^)	0.046 (>0.05)	−0.054 (>0.05)	0.048 (>0.05)
CSF Aβ42 (pg mL^−1^)	−0.587 (*p* < 0.01) *	0.092 (>0.05)	−0.347 (<0.01) *
CSF t-Tau (pg mL^−1^)	0.352 (<0.02) *	0.19 (>0.05)	0.351 (<0.01) *
CSF p-Tau181 (pg mL^−1^)	0.414 (<0.01) *	−0.097 (>0.05)	0.337 (<0.01) *
CSF NfL (pg mL^−1^)	0.357 (>0.05)	0.622 (<0.01) *	0.26 (>0.05)
CSF Aβ42/Aβ40	−0.543 (<0.01) *	0.213 (>0.05)	−0.396 (<0.01) *
CSF t-Tau/Aβ42	0.471 (<0.01) *	0.006 (>0.05)	0.470 (<0.01) *

* = statistically significant *p*-value (<0.05).

**Table 4:** Regression coefficients for the variables in each model.

Coefficients	Model 1	Model 2	Model 3
Intercept	2.530	2.644	2.150
Sex	−0.051	−0.196	−0.066
Age	+0.029	+0.022	+0.026
p-Tau181	+0.023	+0.027	+0.020
GFAP	+0.002	+0.003	+0.002
NfL	+0.023	+0.023	+0.018

Model 1: SCI vs. MCI-AD; Model 2: SCI vs. AD dementia; Model 3: SCI vs. AD (MCI + dementia).

**Table 5:** Diagnosis indexes for each developed model.

	Model 1	Model 2	Model 3
AUC (CI (95%))	0.9256 (0.85–0.98)	98.7 (94–100)	93.5 (87–98)
Sensitivity (%, CI (95%))	89.8 (64–98)	88.9 (83–100)	86.2 (66–98)
Specificity (%, CI (95%))	82.3 (72–100)	100 (81–100)	88.2 (75–100)
Accuracy (%, CI (95%))	87.9 (73–95)	96.1 (88–100)	86.7 (73–96)

Model 1 = SCI vs. MCI-AD; Model 2 = SCI vs. AD dementia; Model 3 = SCI vs. AD (MCI and dementia); CI: Confidence interval.

## Data Availability

Data are available on request from the corresponding author.
